# Clustering based on renal and inflammatory admission parameters in critically ill patients admitted to the ICU

**DOI:** 10.1371/journal.pone.0307938

**Published:** 2024-11-01

**Authors:** Olivier Mascle, Claire Dupuis, Marina Brailova, Benjamin Bonnet, Audrey Mirand, Romain Chauvot De Beauchene, Carole Philipponnet, Mireille Adda, Laure Calvet, Lucie Cassagnes, Cécile Henquell, Vincent Sapin, Bertrand Evrard, Bertrand Souweine

**Affiliations:** 1 CHU de Clermont-Ferrand, Service de Médecine Intensive et Réanimation, Clermont-Ferrand, France; 2 Unité de Nutrition Humaine, INRAe, CRNH Auvergne, Université Clermont Auvergne, Clermont Ferrand, France; 3 CHU de Clermont-Ferrand, Service de Biochimie Médicale, Clermont-Ferrand, France; 4 CHU de Clermont-Ferrand, Service d’Immunologie, Clermont-Ferrand, France; 5 Laboratoire d’Immunologie, ECREIN, UMR1019 UNH, UFR Médecine de Clermont-Ferrand, Université Clermont Auvergne, Clermont-Ferrand, France; 6 CHU de Clermont-Ferrand, 3IHP, Service de Virologie, Clermont-Ferrand, France; 7 UMR CNRS 6023 LMGE, Université Clermont Auvergne, Clermont-Ferrand, France; 8 CHU de Clermont-Ferrand, Service de Radiologie, Clermont-Ferrand, France; 9 CHU de Clermont-Ferrand, Service de Néphrologie, Clermont-Ferrand, France; Stellenbosch University, SOUTH AFRICA

## Abstract

**Introduction:**

The COVID-19 pandemic has been associated with significant variability in acute kidney injury (AKI) incidence, leading to concerns regarding patient heterogeneity. The study’s primary objective was a cluster analysis, to identify homogeneous subgroups of patients (clusters) using baseline characteristics, including inflammatory biomarkers. The secondary objectives were the comparisons of MAKE-90 and mortality between the different clusters at three months.

**Methods:**

This retrospective single-center study was conducted in the Medical Intensive Care Unit of the University Hospital of Clermont-Ferrand, France. Baseline data, clinical and biological characteristics on ICU admission, and outcomes at day 90 were recorded. The primary outcome was the risk of major adverse kidney events at 90 days (MAKE-90). Clusters were determined using hierarchical clustering on principal components approach based on admission characteristics, biomarkers and serum values of immune dysfunction and kidney function.

**Results:**

It included consecutive adult patients admitted between March 20, 2020 and February 28, 2021 for severe COVID-19. A total of 149 patients were included in the study. Three clusters were identified of which two were fully described (cluster 3 comprising 2 patients). Cluster 1 comprised 122 patients with fewer organ dysfunctions, moderate immune dysfunction, and was associated with reduced mortality and a lower incidence of MAKE-90. Cluster 2 comprised 25 patients with greater disease severity, immune dysfunction, higher levels of suPAR and L-FABP/U Creat, and greater organ support requirement, incidence of AKI, day-90 mortality and MAKE-90.

**Conclusions:**

This study identified two clusters of severe COVID-19 patients with distinct biological characteristics and renal event risks. Such clusters may help facilitate the identification of targeted populations for future clinical trials. Also, it may help to understand the significant variability in AKI incidence observed in COVID-19 patients.

## Introduction

Over the last few years the SARS-CoV2 pandemic has been one of the main causes of intensive care unit (ICU) admissions for acute hypoxemic respiratory failure (AHRF) and of ICU mortality [1].

The development of COVID-19-related acute kidney injury (C-AKI) has been associated with substantial morbidity and mortality similar to that observed in other severe infections requiring ICU admission [2–6]. The pathophysiology of C-AKI involves regional and systemic inflammation and immune responses, microvascular thrombosis, endothelial and tubular injury and, to a lesser extent direct viral injury [7, 8]. Several risk factors have been reported with the development of C-AKI including premorbid factors (such as diabetes, chronic kidney disease, obesity, hypertension, male gender and increasing age), the severity of COVID-19 on ICU admission (wave of COVID-19 pandemic, hypoxia intensity, associated organ dysfunction, elevated serum values of systemic inflammation and of organ damage), and patient management (nephrotoxin exposure, organ supports, excessive fluid overload or diuretic-induced hypovolemia) [9].

The reported incidence of C-AKI in ICU varies widely in the literature, ranging from 20% to 70% [9]. As is that of the long term complications of C-AKI such as chronic kidney disease (CKD) and requirement for extended renal replacement therapy (RRT) [4, 10]. The differences observed in the results of these studies can be partly explained by a great heterogeneity in the study populations. Cluster analysis is one technique to investigate this variable.

Cluster analyses are statistical methods that can identify homogeneous subgroups of patients [11] on the basis of their characteristics. Such approaches have already been used to identify subgroups of patients in sepsis or with acute respiratory failure [12, 13] but very few studies have assessed the heterogeneity of patients admitted to the intensive care unit (ICU) for severe COVID-19 [14, 15], and none for C-AKI.

Several biomarkers have been tested during the last decade to predict the development of AKI and mortality in ICU patients including urinary tissue inhibitor of metalloproteinase 2 (TIMP-2), insulin-like growth factor-binding protein 7 (IGFBP-7) [16, 17], soluble receptor for advanced glycation end products (sRAGE) [18], soluble urokinase plasminogen activator receptor (suPAR) [19], and liver-type fatty acid-binding protein (L-FABP) [19]. Recent studies have reported this kind of relationship in COVID-19 patients with C-AKI development or outcome but focused on a single or small number of biomarkers [20–22]. In severe COVID-19, an oversized dysregulation of the inflammation-immunosuppression balance, evaluated by biomarkers such as serum interleukin 6 (IL-6), monocyte human histocompatibility leukocyte antigen expression rate mHLA-DR, and the IL-6:mHLA-DR ratio have been associated with worst outcome, including C-AKI and mortality [23, 24].

The study’s primary objective was a cluster analysis, to identify homogeneous subgroups of patients (clusters) using baseline characteristics, including inflammatory biomarkers. The secondary objectives were the comparisons of MAKE-90 and mortality between the different clusters at three months.

## Materials and methods

### Study design

This study was a retrospective analysis of data prospectively collected. It was a sub-study of Calvet and al. study [25]. It was a single-center observational study performed in the medical ICU of the university hospital of Clermont-Ferrand, France. All consecutive adult patients aged ≥18 years admitted between March 20, 2020 and February 28, 2021 with a positive SARS-CoV-2 polymerase chain reaction test result, pneumonia confirmed by computed tomography, and hypoxemia requiring>6 L/min supplemental oxygen were enrolled. Exclusion criteria were patients referred from another ICU, patients with chronic kidney disease stages 4 and 5, kidney transplant recipients, and patients who declined to participate. Patients with an ICU stay of less than 72 hours were also excluded, since they could not be classified according to our definitions. If a patient was readmitted to the ICU several times during the study period, only the first ICU admission was taken into account.

### Ethics

The study received approval from the ethics committee of the French Intensive Care Society (CE-SRLF21-21) in accordance with French law the 07/03/2021 and Helsinki Declaration of 1975 for “"Insuffisance rénale aiguë et maladie rénale aiguë chez les patients admis en réanimation pour COVID, étude rétrospective d’une cohorte observationnelle (étude SARCOVAKI)"”. Participants provided informed consent verbally after receiving the welcome booklet at ICU admission. Data were accessed for research purpose the 02/06/2023 after being fully anonymized.

### Data collection

All data were extracted from medical records and electronic patients’ charts. Blood sampling, urine analyses and routine biological tests were performed on the day of admission according to standard laboratory protocols.

### Laboratory results

Plasma suPAR levels were measured using commercial Enzyme-linked Immunosorbent Assay (ELISA) kits (suPARnostic® AUTO Flex ELISA Virogates, Copenhagen, Denmark). Plasma sRAGE was measured over human ELISA kits (PROTEIN SIMPLE–BioTechne, Minneapolis, MN, USA). L-FABP concentrations were measured in urine using the ELISA kit from Hycult Biotech (HK404-01, Uden, Netherlands). TIMP-2 and IGFBP7 were measured in urine with the NephroCheckTM Test (Astute Medical, San Diego, CA, USA). The NephroCheck Test is a point-of-care test which was developed to simultaneously measure urine [TIMP-2]*[IGFBP7], whereas [TIMP2]*[IGFBB7] indicates the multiplication of both biomarkers. The quantitative expression of mHLA-DR was determined with the anti-HLA-DR/anti-Monocyte QuantiBRITE assay (BD Biosciences, San Jose, CA, USA). Human pro-inflammatory cytometric bead array (CBA) was used. The human pro-inflammatory cytokine kit simultaneously detects IL-6, IL-10 and CXCL8 cytokines in a single sample.

### Definitions

Phenotypes of AKI were defined according to the Kidney Disease Improving Global Outcomes (KDIGO) guidelines [26], using only the serum creatinine (SCr) component, and early-onset AKI as an AKI occurring within 7 days. “Baseline SCr” was the best outpatient SCr value between 7 and 365 days before ICU admission or, if unavailable, was estimated using the Modification of Diet in Renal Disease (MDRD) equation [27]. Early-onset AKI (EO-AKI) was defined as AKI occurring within 7 days after ICU admission: only the first EO-AKI episode was taken into account in the study. Patients were stratified according to the highest AKI stage attained during this first episode. EO-AKI recovery was based on a sustained (≥48hours) and complete reversal of AKI by KDIGO criteria and therefore a minimum of 48 hours of renal recovery was necessary to separate two distinct AKI episodes. EO-AKI was classified as transient, persistent and AKD according to the *Acute Dialysis Quality Initiative* [28]. “Transient” AKI was defined as renal recovery within 48 h of AKI onset, and “persistent” AKI as renal recovery occurring ≥3 days and <7 days of EO-AKI onset. Acute kidney disease (AKD) was characterized when AKI stage 1 or greater persisted ≥7 days after EO-AKI onset [28] (Annex 2). Chronic kidney disease (CKD) staging was defined according to the KDIGO criteria [27].

MAKE-90 was a composite outcome of death, dialysis dependence, or glomerular filtration rare (eGFR) < 60 mL/min observed 90 days after ICU admission [28–30]. SCr at 90 days was retrieved from the medical health records and if unavailable the patient’s general practitioner was contacted.

### Objectives

The study’s primary objective was a cluster analysis, to identify homogeneous subgroups of patients (clusters) using baseline characteristics, including inflammatory biomarkers. The secondary objectives were the comparisons of MAKE-90 and mortality between the different clusters at three months.

### Statistical analysis

Baseline characteristics were reported as median [interquartile range] and n (%) for quantitative and categorical variables, respectively. Quantitative variables were compared with nonparametric tests, the Mann–Whitney test or the Kruskal–Wallis test, as appropriate. Categorical variables were compared with Pearson’s Chi-square test or Fisher’s exact test, as appropriate.

A two-step clustering approach was used to reduce the dimensionality of the dataset and to perform hierarchical clustering. This approach, called hierarchical clustering on principal components (HCPC), was performed using the factoMineR package in R. We first performed a principal component analysis of clinical characteristics (age and body mass index (BMI)), and biological features, (IL-10, IL-1, IL-6, TNF, mHLA-DR, [TIMP-2] *[IGFBP7], L-FABP, RAGE, suPAR, creatinine, PCT, CRP, neutrophils, lymphocytes, D-dimer and ferritin). Variables were standardized as they were measured in different units. The HCPC procedure makes it possible, after the hierarchical clustering step is performed, to choose the number of clusters based on the hierarchical tree and to perform a K-means clustering to improve the initial partition obtained from the hierarchical clustering [31]. Ward’s criterion and a Euclidean metric were used for agglomerative hierarchical clustering.

We then described and compared variables (baseline characteristics, laboratory features, radiological findings and outcomes) within and between clusters. The clustering approach identified three clusters but because cluster 3 comprised only two patients the following analyses focused only on clusters 1 and 2.

Using a random forest approach, we determined the variables that best identified clusters 1 and 2. The random forest method is a type of decision-tree learning algorithm that is able to address nonlinear relationships and complex interactions between potential explanatory variables and to rank the relative importance of each factor in predicting membership of clusters 1 and 2. The random forest analysis was conducted with the random Forest package in R.

A decision tree was then constructed to predict the belongings to one cluster using rpart package in R.

The probability for each variable of belonging to a cluster 1 or 2 was also assessed by odds ratios, determined by univariable logistic regression.

Finally, using univariate logistic regression, we tested the associations between clusters 1 and 2 and main outcomes including ICU and day-90 mortality and MAKE-90. Co-variates were dichotomized with the usual cut-off or their medians in our cohort.

A p-value lower than 0.05 was considered significant. Missing data were imputed by multiple imputation using Proc MI from SAS software. Only the first imputed dataset was considered for the clustering approach and other analyses. All statistical analyses were performed with SAS software, Version 9.4 (SAS Institute, Cary, NC) and R (version 3.6.3).

## Results

### Study population

Characteristics of the studied population are shown in Table 1. The study sample comprised 149 COVID-19 patients EO-AKI was observed in 46 (31%) patients and RRT was needed in 21 (14%) patients. ICU, hospital and 90-day mortality were 30%, 35% and 37%, respectively.

**Table 1:** Description of the included patients and comparison between clusters 1 and 2.

Parameter	All	Cluster 1	Cluster 2	pval 1|2
**Number of patients**	149	122	25	.
**Age, yr**	71.4 [64.4; 76.2]	70.95 [64.5; 75.8]	74.59 [59.9; 79.5]	0.25
**Sex, no (%), men**	108 (72.4)	88 (72.131)	19 (76)	0.69
**BMI, kg/m^2^**	28.6 [25.4; 32]	28.8 [25.7; 32.0]	27.2 [23.4; 32.3]	0.25
**Comorbidities**				
**BMI > 30 kg/m^2^**	67 (45)	56 (45.902)	10 (40)	0.59
**Cardio-vascular disease**	27 (18.2)	20 (16.393)	7 (28)	0.17
**Chronic respiratory disease**	7 (4.6)	7 (5.738)		0.22
**Immunosuppression§**	24 (16.2)	18 (14.754)	5 (20)	0.51
**Diabetes mellitus**	24 (16.2)	18 (14.754)	5 (20)	0.51
Time from 1^st^ symp to ICU admission	9 [7; 11]	9 [7; 11]	11 [7; 13]	0.12
**Converting Enzyme Inhibitors**	4 (2.6)	3 (2.459)	1 (4)	0.67
**Diarrhea**	11 (7.4)	11 (9.016)		0.12
**Lopinavir Ritonavir**	2 (1.4)	1 (0.82)	1 (4)	0.21
**Remdesivir**	47 (31.6)	43 (35.246)	3 (12)	0.02
**Corticosteroids**	130 (87.2)	111 (90.984)	17 (68)	<0.01
**Antimicrobial therapy**	36 (24.2)	25 (20.492)	10 (40)	0.04
**Aminoglycosides**	2 (1.4)	1 (0.82)	1 (4)	0.21
**Vancomycine**	2 (1.4)	1 (0.82)	1 (4)	0.21
**Diuretics**	132 (88.6)	109 (89.344)	21 (84)	0.43
**Extent of COVID lesion on CT scan**	47 [33; 63]	46.5 [30; 60]	58 [46; 70]	0.02
*Type of variant- Clade|Lineage|WHOlabel*				
**19B|A**	1 (0.67)	0	1 (4.55)	
**20A|B.1.160|EU2**	59 (39.60)	47 (41.59)	10 (45.46)	0.51
**20A|otherslineagesthan**	13 (8.72)	11 (9.74)	2 (9.09)	
**20C|B.1.367**	1 (0.67)	1 (0.89)		
**20D|B.1.1.16**	1 (0.67)	1 (0.89)		
**20E|B.1.177|EU1**	10 (6.7)	8 (7.08)	2 (9.09)	
**20I|B.1.1.7|Alpha**	2 (1.34)	2 (1.77)		
**nongenotyped**	43 (38.053)	7 (31.82)		
*During ICU first two days*				
**SAPS II**	35 [29; 44]	35 [29; 42]	42 [31; 49]	0.03
**SOFA**	4 [3; 5]	4 [3; 5]	5 [4; 8]	<0.01
**PaO2/FiO2**	101 [70; 232]	106 [76; 280]	76 [58; 104]	0.01
**Vasopressors**	18 (12)	10 (8.197)	7 (28)	<0.01
**Invasive mechanical ventilation**	20 (13.4)	12 (9.836)	7 (28)	0.01
**Renal replacement therapy**	2 (1.4)		2 (8)	<0.01
**Pneumonia on admission**	11 (7.4)	9 (7.377)	1 (4)	0.54
**Bacteriemia**	4 (2.6)	1 (0.82)	3 (12)	<0.01
*Characteristics of AKI during ICU stay*				
**No AKI**	103 (69.1)	92 (75.41)	11 (44)	<0.01
**Transient AKI**	20 (43.4)	14 (11.475)	6 (24)	.
**Persistant AKI**	12 (26)	6 (4.918)	5 (20)	.
**Acute kidney disease**	14 (30.4)	10 (8.197)	3 (12)	.
**No AKI**	103 (69.2)	93 (76.23)	10 (40)	<0.01
**Kdigo 1**	27 (18.2)	19 (15.574)	8 (32)	.
**Kdigo 2**	9 (6)	4 (3.279)	4 (16)	.
**Kdigo 3**	10 (6.8)	6 (4.918)	3 (12)	.
*During the whole ICU stay*				
**Vasopressors**	50 (33.6)	35 (28.689)	13 (52)	0.02
**Invasive mechanical ventilation**	50 (33.6)	36 (29.508)	12 (48)	0.07
**ECMO**	1 (0.6)	1 (0.82)		0.65
**Invasive mechanical ventilation duration**	0 [0; 5]	0 [0; 2]	0 [0; 11]	0.05
**Pulmonary embolism**	8 (5.4)	7 (5.738)	1 (4)	0.73
**Ventilator associated pneumoniae**	18 (12)	13 (10.656)	5 (20)	0.19
**Renal replacement therapy**	21 (14)	15 (12.295)	5 (20)	0.31
**Decision not to intubate during ICU Stay**	25 (16.8)	16 (13.115)	7 (28)	0.06
**Length of ICU stay, d (IQR)**	7 [5; 12]	7 [5; 11]	9 [6; 15]	0.09
**Death in ICU**	45 (30.2)	30 (24.59)	13 (52)	<0.01
**Length of Hospital Stay, d (IQR)**	15 [10; 24]	16 [10; 24]	15 [9; 21]	0.63
**In-hospital death**	52 (34.8)	35 (28.689)	15 (60)	<0.01
**RRT at hospital discharge (missing = 59)**	1 (1.2)	9 (11.538)	1 (12.5)	0.94
**GFR < 60 mL/min at 90 days (missing = 63)**	10 (11.6)	1 (1.235)		0.74
**RRT at 90 days (missing = 62)**	0	0	0	
**Death at 90 days**	55 (37)	37 (30.328)	16 (64)	<0.01
**MAKE-90**	65 (43.6)	46 (37.705)	17 (68)	<0.01

AKI: acute kidney injury, BMI: body mass index, GFR: glomerular filtration rate, ICU: intensive care unit, IMV: invasive mechanical ventilation, KDIGO: Kidney Disease Improving Global Outcomes, LOS: length of stay, MAKE: major adverse kidney event, RRT: renal replacement therapy, SAPS II: simple acute physiology score II, SOFA: sequential organ failure assessment.

§Aplasia (lymphocytes < 1000/mm3); or corticosteroids (if treatment duration >1 month or if treatment amount >2mg/kg regardless of duration); or HIV (positive serology); AIDS (positive HIV serology and clinical complications: pneumocystis pneumonia, Kaposi’s sarcoma, tuberculosis, toxoplasmosis

### Identification of clusters

**Fig 1:**
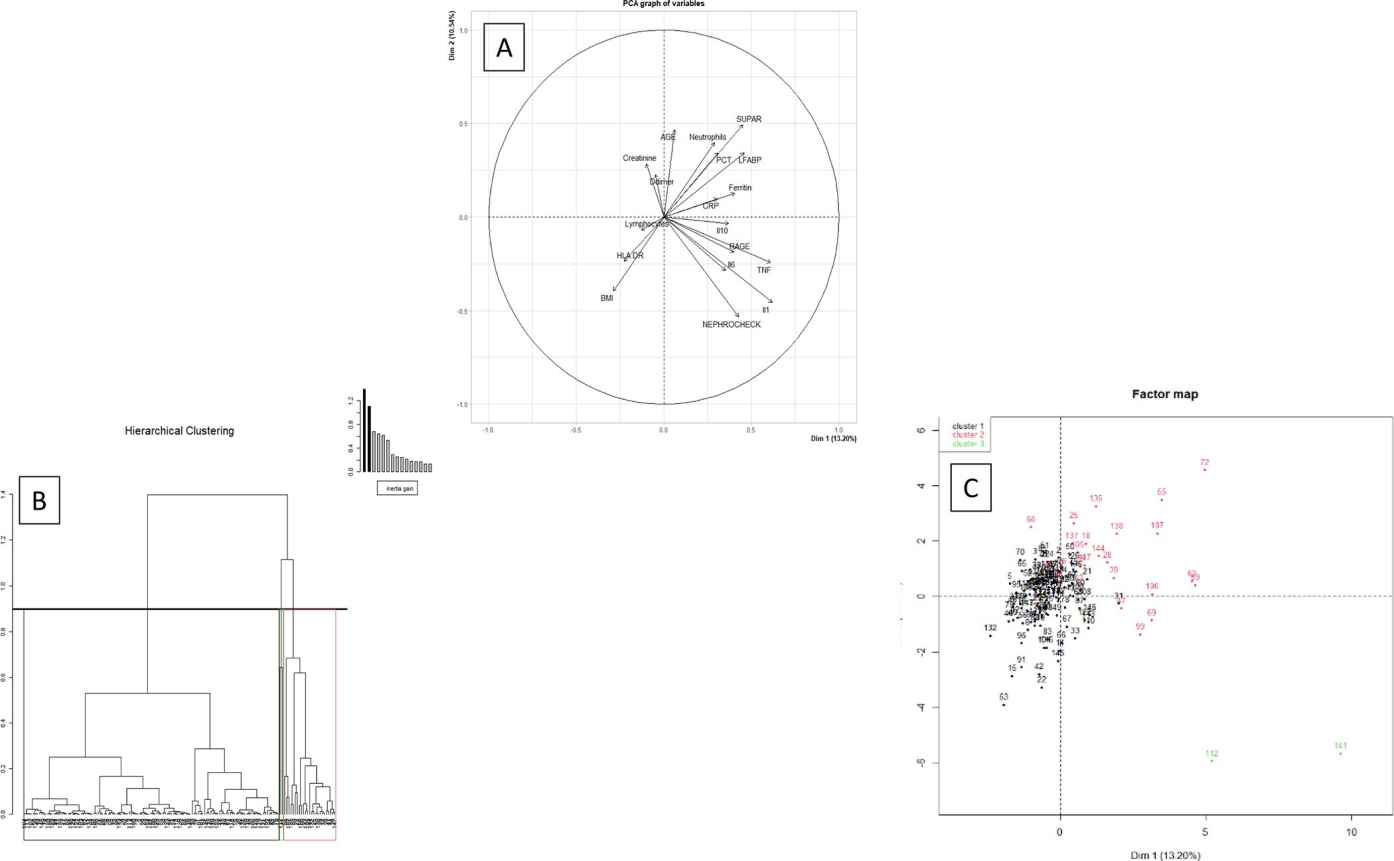
Hierarchical clustering on principal components using principal component analysis. A: Graph of variables: projection on the first and second dimensions of the covariates used for the clustering. B: Dendrogram of ascending hierarchical clustering analysis. Dendrogram obtained after application of hierarchical clustering analysis. The vertical axis of the dendrogram represents the distance between clusters. The horizontal vertical axis represents the patients and clusters. Each junction between two clusters is represented on the graph by the split of a vertical line into two vertical lines. The vertical position of the split, shown by the short horizontal bar gives the distance between the two clusters. The red line shows the cut level that determines the number of clusters. C: Representation of the patients into the first two dimensions. Axes correspond to the first and second dimension of MCA. All patients were represented by their individual coordinates in these dimensions. MCA: multiple correspondence analysis, HC: hierarchical clustering, all patients were represented by their individual coordinates in these dimensions.

The contributions of the variables to the construction of the first two CPA dimensions are presented in Fig 1A–1C illustrates the projection of patients in the first two dimensions of PCA. The HCPC finally identified three clusters (Fig 1C). Clusters 1, 2 and 3 comprised 122, 25 and 2 patients, respectively.

### Description of clusters

The two patients of Cluster 3 were very acutely ill. They were admitted to ICU with multiple organ failure and extremely high immune dysregulation. They died 7 and 13 days after withdrawal of life-sustaining treatment (S1 and S2 Tables in S1 Appendix). Because of the small sample size of Cluster 3, the following results focused on Clusters 1 and 2 (Tables 1 and 2). On admission, compared to Cluster 1 patients, those of Cluster 2 were characterized by a more profound immune dysregulation with higher neutrophil counts, higher serum levels of fibrinogen, C-reactive protein, ferritin, IL-1b, IL-6, IL-8, IL-10, IL-12, a lower expression of mHLA-DR, and greater mHLA-DR/Il6 values. They had a higher SOFA score and SAPS 2, a greater extent of CT-scan pulmonary lesions, a more profound hypoxemia, a greater need for IMV, a less frequent administration of remdesivir and of steroids, and higher values of L-FABP/U Creat and SuPAR. During the ICU stay, Cluster 2 patients received more frequently IMV and vasopressors, and developed more frequently EO-AKI than those in Cluster 1. ICU mortality, day-90 mortality and MAKE-90 were higher in Cluster 2 than in Cluster 1 patients, 52% vs 25% (p = 0.006), 64% vs 30% (p = 0.003), and 68% vs 38% (p = 0.005), respectively. The predominant type of SARS-CoV2 variant in both cluster was 20A|B.1.160|EU2 (42% in cluster 1 and 45% in cluster 2 (p = 0.81)).

**Table 2:** Biological characteristics in the whole cohort and comparisons between Clusters 1 and 2.

	All	Cluster 1	Cluster 2	pval 1|2
**Number of patients**	149	122	25	
Neutrophils^a^	7 [5; 10.6]	6.47 [4.76; 9.32]	12.82 [9.46; 17.02]	<0.01
Lymphocytes^a^	0.6 [0.4; 1]	0.64 [0.46; 0.87]	0.82 [0.47; 1.11]	0.09
Procalcitonin^b^ *(miss = 6)*	0.2 [0.2; 0.6]	0.2 [0.11; 0.38]	0.58 [0.26; 1.79]	<0.01
C Reactive Protein^c^ *(miss = 32)*	128 [74; 176]	129 [82; 143]	129 [110; 217]	0.02
D-dimers^d^	1252 [786; 2246]	1211 [754; 2012]	1916 [953; 3381]	0.12
Fibrinogen^e^ *(miss = 1)*	7.4 [6.2; 8]	7.1 [6.1; 7.9]	8 [7.5; 8.8]	<0.01
Ferritin^b^ *(miss = 5)*	1126 [635; 1944.6]	1029.5 [524; 1677]	1466 [789; 2135]	0.02
IL-10^f^ *(miss = 1)*	3.4 [2; 5.8]	3.2 [1.85; 5.1]	6.3 [4.2; 10.9]	<0.01
IL-12^f^ *(miss = 1)*	0.2 [0; 1.4]	0.2 [0; 1]	1.13 [0; 2]	0.01
IL-1b^f^ *(miss = 1)*	0.2 [0; 1]	0 [0; 1]	0.5 [0; 1.83]	0.09
IL-6^f^ *(miss = 1)*	34 [10.8; 67.4]	24.75 [9.1; 57.8]	82.6 [44.9; 121.3]	<0.01
IL-8^f^ *(miss = 1)*	25.4 [16.6; 36.8]	23.05 [15.3; 32.99]	37.02 [26.1; 53.4]	<0.01
TNF alpha^f^ *(miss = 1)*	0.2 [0; 1.2]	0 [0; 1.1]	1 [0.2; 1.4]	<0.01
mHLA DR^g^ (miss = 13)	9487 [6472.6; 13337.6]	9794.5 [7003; 13491]	7774 [4881; 9571]	0.01
**mHLA DR/Il6 (miss = 13)**	3.6 [1.2; 7.8]	3.09 [0.78; 6.3]	9.2 [5.79; 19.76]	<0.01
[TIMP-2] × [IGFBP7] *(miss = 2)*	0.4 [0.2; 1]	0.43 [0.19; 1.02]	0.59 [0.29; 1.23]	0.377
L-FABP^d^/U Creat^i^	190 [80.4; 470]	176.65 [70.8; 326.6]	402.57 [184.3; 1203]	<0.01
sRAGE^h^ *(miss = 2)*	2906 [1460; 7156]	2887.5 [1449; 6286]	2906 [1823; 10352]	0.47
suPAR^d^ *(miss = 2)*	6.8 [5.4; 9]	6.27 [5.19; 8.09]	10.05 [8.15; 14.41]	<0.01

a: G/L, b: μg/L, c: mg/L, d: ng/mL, e: g/L, f:pg/L, g:HLA-DR count on monocyte, h: pg/mL, i:mmol/L; IGFBP-7: insulin-like growth factor-binding protein 7, IL: interleukin, L-FABP: liver fatty acid binding protein, sRAGE: soluble receptor of advanced glycation end products, suPAR: soluble urokinase plasminogen activator receptor, TIMP-2: tissue inhibitor of metalloproteinase 2, TNF: tumor necrosis factor

**Fig 2:**
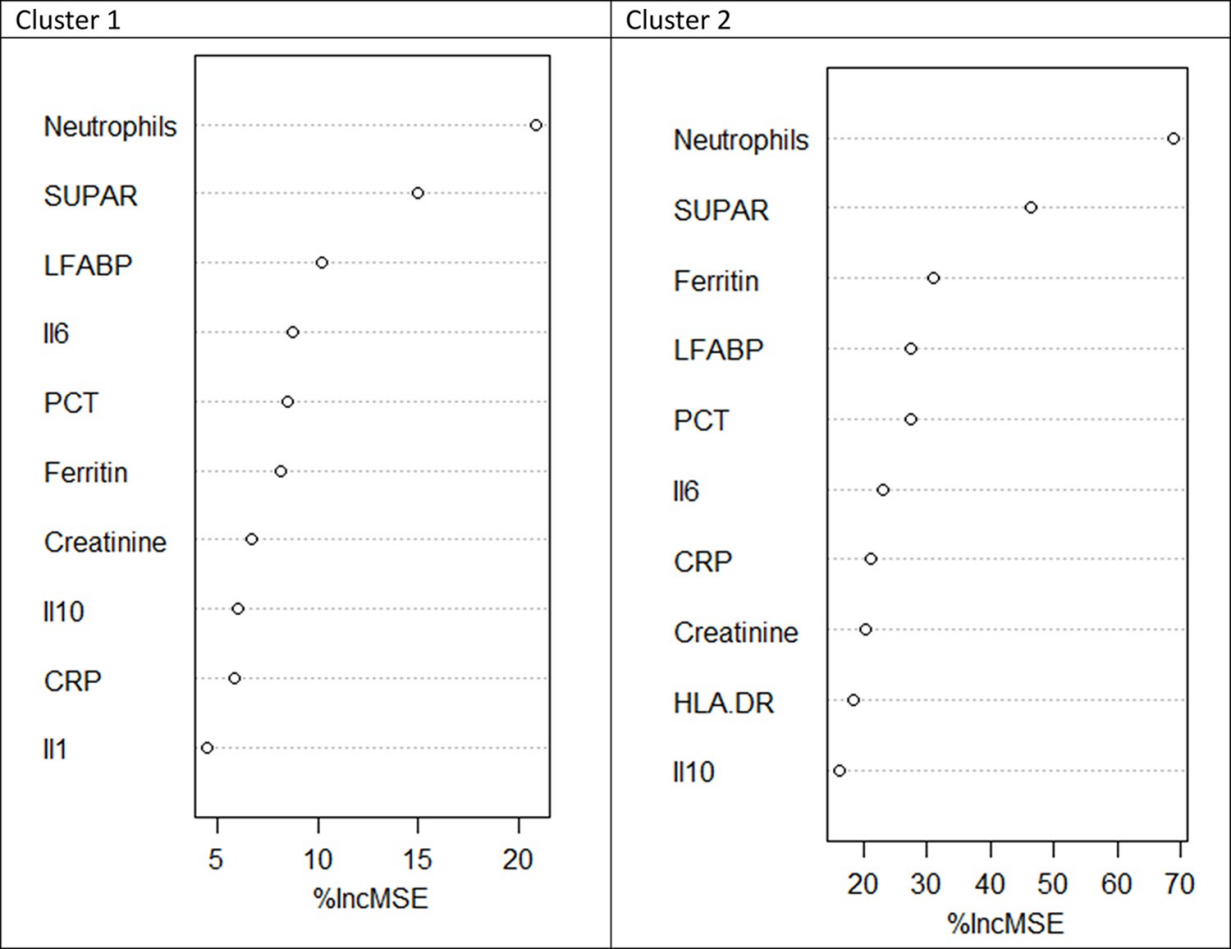
Prediction to belong to Cluster 1 or Cluster 2 using random forest algorithm. %Inc MS:, per cent increase in mean squared error, CRP: C reative protein, Il: interleukin,; LFABP: liver fatty acid binding protein, MCA: multiple correspondence analysis, mHLA DR, Monocytic human leukocyte antigen-DR, PCT: procalcitonin, SUPAR: soluble urokinase plasminogen activator receptor, TNF: tumor necrosis factor.

**Fig 3:**
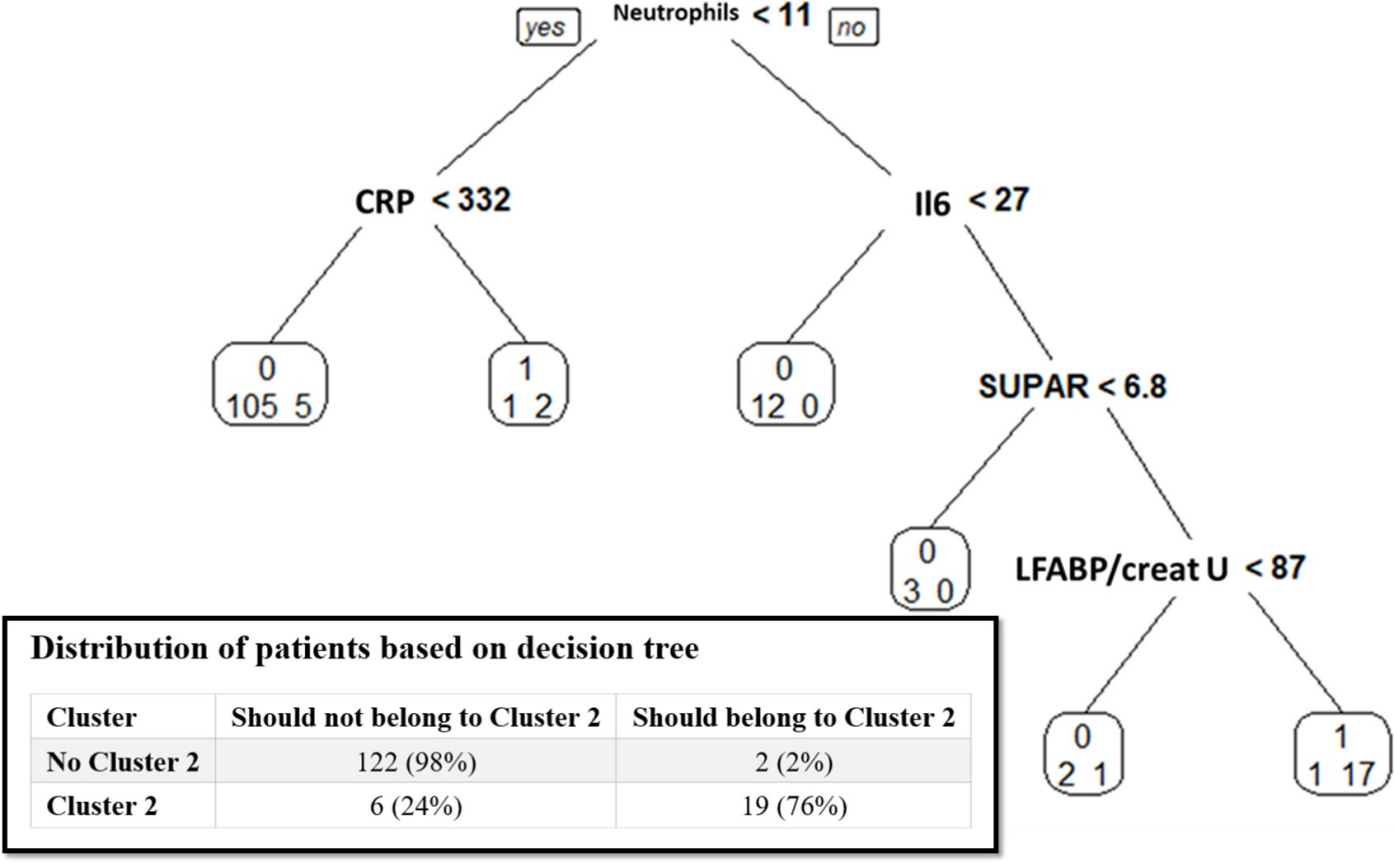
Prediction to belong to Cluster 2 using classification and regression tree analysis. Tree is built such as 0: should not belong to cluster 2, 1: should belong to cluster 2; and below: left number actual patients in the studied population who did not belong to cluster 2; right number: actual patients in the studied population who belonged to Cluster 2. CRP: C-reactive protein (in mg/L), creat U: urinary creatinine (in mmol/L), Il, interleukin (in pg/L,; LFABP, liver fatty acid binding protein (in ng/mL,; SUPAR, soluble urokinase plasminogen activator receptor (in ng/mL) The binary tree was built in the training set using Breiman methods with Rpart package version 4.1–10, R version 3.1.0. The structure is similar to a real tree, from the bottom up: there is a root, where the first split happens. After each split, two new nodes are created. Each node contains only a subset of the patients. The partitions of the data, which are no longer split, are called terminal nodes or leafs. The second stage of the procedure consists in pruning the tree using cross-validation. Pruning means to shorten the tree, which makes trees more compact and avoids over-fitting to the training data. Each split is examined if it makes a reliable improvement. The six variables used by the binary tree are neutrophils, CRP, IL-6, SUPAR and LFABP/urinary creatinine ratio. The accuracy of the binary tree evaluated in the training dataset is given in Table 4.

The distribution of variables best able to identify membership of Cluster 1 and Cluster 2 determined by logistic regression and random forest approach are shown in Table 3 and Fig 2. A decision tree for identifying patients belonging to Cluster 2 was developed using neutrophil count, CRP and IL-6 serum levels and L-FABP/urinary creatinine ratio. The sensitivity and specificity of this decision tree tested on the overall population to identify Cluster 2 patients was 76%and 98% respectively (Fig 3).

**Table 3:** Factors associated with Cluster 1 or 2.

Risk factor	OR	IC95	pvalue	OR	IC95	pvalue
	**Cluster 1**			**Cluster 2**		
**Symptoms to ICU>10 days**	0.49	[0.21; 1.14]	0.10	2.45	[1.02; 5.87]	0.04
**Age > 70 years**	0.78	[0.34; 1.83]	0.57	1.32	[0.55; 3.16]	0.54
**Sex (Male)**	0.91	[0.35; 2.34]	0.84	1.25	[0.46; 3.38]	0.67
**BMI > 30 kg/m^2^**	1.23	[0.53; 2.88]	0.63	0.78	[0.33; 1.88]	0.58
**Comorbidities**	0.53	[0.23; 1.26]	0.15	1.60	[0.67; 3.83]	0.29
**Immunodepression**	0.61	[0.21; 1.71]	0.34	1.38	[0.46; 4.13]	0.56
**Lopinavir ritonavir**	0.21	[0.01; 3.55]	0.28	5.13	[0.31; 84.8]	0.25
**Remdesivir**	3.13	[1.02; 9.64]	0.05	0.25	[0.07; 0.88]	0.03
**Corticosteroids**	4.25	[1.51; 11.93]	<0.01	0.21	[0.07; 0.59]	<0.01
**Antimicrobial therapy**	0.37	[0.15; 0.91]	0.03	2.51	[1.01; 6.24]	0.05
**Aminosides**	0.21	[0.01; 3.55]	0.28	5.13	[0.31; 84.8]	0.25
**Vancomycin**	0.21	[0.01; 3.55]	0.28	5.13	[0.31; 84.8]	0.25
**Diuretics**	1.46	[0.44; 4.88]	0.54	0.61	[0.18; 2.07]	0.43
**SOFA > 4**	0.38	[0.16; 0.92]	0.03	2.23	[0.92; 5.43]	0.08
**Vasopressors**	0.21	[0.07; 0.61]	<0.01	3.99	[1.37; 11.65]	0.01
**IMV**	0.26	[0.09; 0.72]	<0.01	3.32	[1.17; 9.44]	0.02
**Pneumonia on admission**	1.00	[0.2; 4.89]	1.00	0.48	[0.06; 3.89]	0.49
**Neutrophils > 8 G/L**	0.13	[0.04; 0.41]	<0.01	9.51	[2.7; 33.42]	<0.01
**Lymphocytes > 0.6 G/L**	1.64	[0.68; 3.94]	0.27	0.57	[0.23; 1.42]	0.23
**CRP > 100 mg/L**	0.36	[0.12; 1.1]	0.07	2.50	[0.8; 7.77]	0.11
**D-Dimers > 1500 μ/L**	0.60	[0.26; 1.39]	0.24	1.66	[0.7; 3.93]	0.25
**Fibrinogen > 8 g/L**	0.23	[0.08; 0.64]	<0.01	5.25	[1.7; 16.18]	<0.01
**Ferritin > 1000 μg/L**	0.55	[0.23; 1.32]	0.18	1.93	[0.78; 4.8]	0.16
**PCT > 0.5 μg/L**	0.22	[0.09; 0.52]	<0.01	4.51	[1.83; 11.13]	<0.01
**Il10 > 4.5 pg/mL**	0.26	[0.1; 0.66]	<0.01	4.24	[1.58; 11.35]	<0.01
**Il12 > 0.2 pg/mL**	0.29	[0.11; 0.73]	<0.01	3.02	[1.18; 7.75]	0.02
**Il1 > 0.2 pg/mL**	0.44	[0.18; 1.03]	0.06	1.94	[0.81; 4.67]	0.14
**Il6 > 34 pg/mL**	0.12	[0.04; 0.37]	<0.01	10.50	[2.98; 36.94]	<0.01
**Il8 > 25 pg/mL**	0.24	[0.09; 0.64]	<0.01	4.70	[1.66; 13.32]	<0.01
**TNF > 0.2 pg/mL**	0.27	[0.11; 0.68]	<0.01	3.23	[1.26; 8.28]	0.01
**mHLA DR > 9500 pg/mL**	2.37	[1; 5.59]	0.05	0.50	[0.21; 1.19]	0.12
**Il6/mHLA DR (x 1000) > 3.6**	0.12	[0.04; 0.37]	<0.01	10.50	[2.98; 36.94]	<0.01
**(TIMP-2) × (IGFBP7) > 0.3**	0.86	[0.35; 2.14]	0.75	1.01	[0.4; 2.54]	0.98
**LFABP/ creat > 190**	0.33	[0.14; 0.82]	0.02	3.23	[1.26; 8.28]	0.01
**sRAGE > 2900 pg/mL**	0.80	[0.35; 1.85]	0.60	1.05	[0.44; 2.48]	0.91
**suPAR > 6.8**	0.05	[0.01; 0.23]	<0.01	17.02	[3.84; 75.43]	<0.01
**Parenchymal lesions > 75%**	0.27	[0.06; 1.29]	0.10	4.09	[0.86; 19.55]	0.08

BMI: Body mass index, CRP: C reative protein, ICU: Intensive care unit, IGFBP-7: insulin-like growth factor-binding protein 7, Il: Interleukin, IM: Invasive mechanical ventilation, LFABP: Liver fatting acid binding protein, mHLA DR, Monocytic human leukocyte antigen-DR, SOFA: Sequential organ failure assessment, sRAGE: Soluble receptor of advances glycation end product, suPAR: Soluble Urokinase Plasminogen Activator Receptor, TIMP-2: Tissue inhibitor of metalloproteinase 2, TNF: Tumor necrosis factor. Immunosuppression: Hematologic malignancy (active or in remission for less than 5 years), hematopoietic stem cell transplant for less than 5 years, active solid cancer, leukopenia < 1 G/L or neutropenia ≤ 0.5 G/L, solid organ transplantation, syndrome acquired immunodeficiency, long-term corticosteroid therapy ≥ 0.5 mg/kg/day of prednisone equivalent for at least 3 weeks, immunosuppressive or immunomodulatory treatment

**Fig 4:**
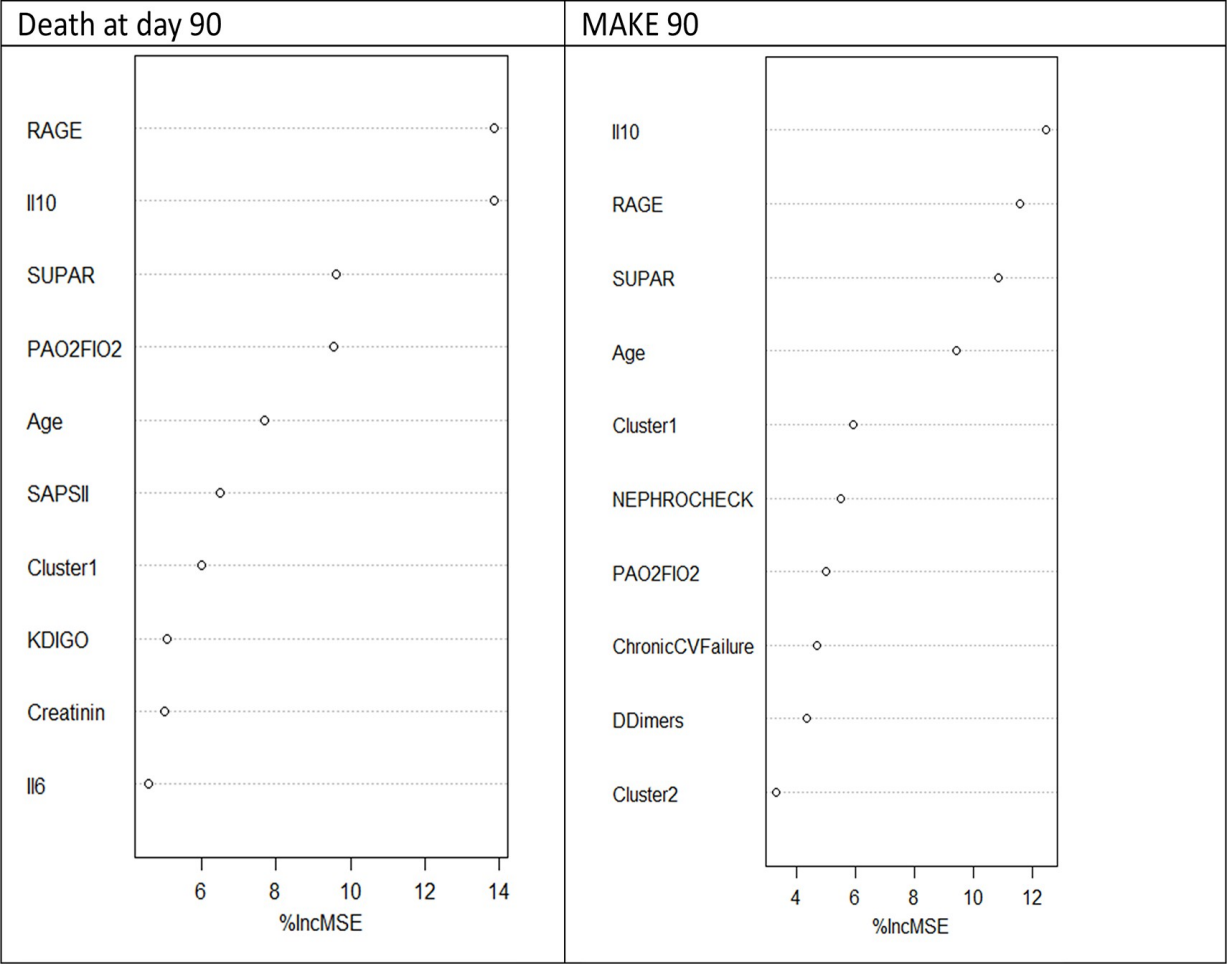
Predictors of death and MAKE 90—Random forest. Contribution of each variable to the risk of death (A) and MAKE 90 (B) %Inc MSE: per cent increase in mean squared error, CV: cardiovascular, Il: interleukin, KDIGO: acute kidney injury classification, LFABP: liver fatty acid binding protein, RAGE: receptor of advanced glycation end product, SUPAR: soluble urokinase plasminogen activator receptor, TNF: tumor necrosis factor, SAPSII: simple acute physiology score II.

Cluster 2 was associated with a higher risk of MAKE-90 (OR 3.36; IC [1.35; 8.4], P <00.1) and Cluster 1 with a lower risk (OR 0.26; IC [0.1; 0.63], P<001). A higher risk of AKI during the ICU stay, and of ICU and day-90 mortality was observed in Cluster 2, and a lower risk in Cluster 1 (Table 4). The factors associated with MAKE-90 and day-90 mortality were determined by random forest (Fig 4). In this last analysis, belonging to Cluster 1 or 2 was not associated with a better prediction for death at day 90 and MAKE-90 than sRAGE, IL-10, suPAR and age.

**Table 4:** Association with AKI on admission and with ICU death, univariate logistic regression.

**Association with AKI on admission**			
**Cluster1**	0.235	[0.09; 0.59]	<0.01
**Cluster2**	4.945	[1.93; 12.67]	<0.01
**Association with ICU mortality**			
**Cluster1**	0.261	[0.11; 0.62]	<0.01
**Cluster2**	3.115	[1.29; 7.52]	0.01
**Association with 90-day mortality**			
**Cluster1**	0.218	[0.09; 0.53]	< .01
**Cluster2**	3.873	[1.57; 9.53]	< .01
**Association with MAKE-90**			
**Cluster1**	0.255	[0.1; 0.63]	< .01
**Cluster2**	3.364	[1.35; 8.4]	< .01

AKI: acute kidney injury, ICU: intensive care unit, MAKE: major acute kidney event.

## Discussion

In this study, we used a clustering approach to investigate renal outcomes among patients admitted to a French ICU with severe COVID-19. Our analysis was based on clinical and biological data available at the time of ICU admission. We successfully distinguished two relatively homogeneous clusters of patients with severe COVID-19, who differed on admission by the severity of illness, the intensity of immune dysregulation, and the risk of MAKE-90, ICU and day-90 mortality. A decision tree built with neutrophils, CRP, IL-6, SuPAR and L-FABP, yielded the identification of Cluster 2 patients, with a good specificity.

Our results warrant several explanations. The demographic characteristics of our patient cohort closely resembled those typically seen in critically ill COVID-19 patients admitted to ICUs [2]. Acute kidney injury (AKI) was observed in 31% of patients during their ICU stay, with MAKE affecting 43.6% at 3 months post-admission aligning with previous research [4]. During follow-up, Nugent et al. also noted a reduced glomerular filtration rate (GFR) in COVID-19 patients who experienced AKI during their hospital stay compared to those who did not, suggesting a delayed or absence of full recovery among the former group [10]. Notably, in our study, COVID-19-related AKI was independently associated with a reduced rate of kidney recovery during outpatient follow-up, emphasizing its clinical significance (adjusted hazard ratio, 0.57; 95% CI, 0.35–0.92).

Cluster 1 encompassed patients who, while sharing similar comorbidities and age to those of Cluster 2, experienced fewer organ dysfunctions, including AKI. Cluster 1 patients had lower ICU, in-hospital, and 90-day mortality rates, and a reduced incidence of MAKE-90. Cluster 1 patients had moderate immune dysfunction, as evidenced by lower concentrations of interleukins (both inflammatory and anti-inflammatory) than those in Cluster 2. Notably, mHLA-DR expression was higher in Cluster 1 than in Cluster 2, suggesting a less pronounced immunoparalysis [32] while levels of IL-6, a proinflammatory cytokine [33], were lower. A greater mHLA-DR expression is known to be associated with better outcomes in COVID-19 [32, 34, 35], and the ratio of IL-6 to mHLA-DR can serve as a valuable marker of severity since ICU admission, as suggested by Bonnet et al. [23]. Almost all the patients in Cluster 1 were receiving steroids, and in several cases treatment was started before ICU admission. Steroid administration may have reduced immune dysfunction, which would explain the better outcomes of the patients in Cluster 1 [36].

Cluster 2 comprised patients with greater illness severity upon ICU admission, as indicated by higher SAPSII and SOFA scores. Cluster 2 patients required more intensive organ support, including vasopressor therapy, IMV and RRT. They had a higher incidence of AKI ICU, and of in-hospital mortality rates and MAKE-90. They also had a more pronounced immune dysfunction on ICU admission, as evidenced by elevated concentrations of inflammatory biomarkers such as neutrophils, fibrinogen, C-reactive protein, procalcitonin, TNF-alpha, interleukins, and IL-6/mHLA-DR ratio. Previous research has linked high plasma concentrations of IL-6 and neutrophils to increased mortality in severe COVID-19 [6], and, similarly, elevated inflammatory parameters are associated with a higher incidence of AKI in COVID-19 (35). As in previously published studies, patients with worse outcomes in our cohort had higher concentrations of anti-inflammatory markers such as IL-10 [23, 24, 37] and lower mHLA-DR expression.

In our study, parameters that proved instrumental in quickly identifying patients belonging to Cluster 2 included neutrophils, CRP, IL-6, suPAR, and the L-FABP/urinary creatinine ratio. Soluble urokinase plasminogen activator receptor (suPAR) is a signaling glycoprotein implicated in the pathogenesis of kidney disease [38] previously associated in sepsis and COVID-19 with an increased risk of AKI and mortality [19, 20, 39–42]. Our results are in agreement since we identified suPAR as a risk factor for MAKE-90. L-FABP, which is primarily found in proximal tubules, has shown promise as a biomarker for various kidney disorders [19]. Although L-FABP levels appear less elevated in COVID-19 than in other pulmonary diseases [43], studies by Tantry et al. [22], and Katagiri et al. [44], in line with our findings, associated higher L-FABP levels with clinical events and prolonged hospitalization.

In our work, the three parameters most strongly associated with MAKE-90 or 90-day mortality were suPAR, sRAGE, and IL-10. The role of sRAGE in AKI development during sepsis remains debated [45]. Brodska et al. reported a correlation between sRAGE concentrations, mortality, and AKI in sepsis patients [18] while Wu et al. found no significant differences in sRAGE concentration between AKI and non-AKI patients [46]. Clinical research indicates that measuring sRAGE in COVID-19 ARDS can serve as a powerful biomarker for managing cytokine storms in lung cells, suggesting it has a role in alleviating respiratory symptoms and early diagnosis of pulmonary complications [47]. Our results further support the involvement of sRAGE in AKI development, which is consistent with known associations between RAGE and COVID-19 mortality. Elevated IL-10 levels in severe COVID-19 have been linked to worse outcomes [23] and AKI [3, 48, 49], as observed in our study.

The value of [TIMP-2] x [IGFBP7], provided by Nephrocheck®, showed a limited correlation with the risk of MAKE-90 in our study. A cutoff of 0.3 has been established to achieve high sensitivity while maintaining acceptable specificity to predict AKI [50, 51]. TIMP-2 and IGFBP-7 are expressed and secreted in various tissues, including the kidneys, where they can induce cell-cycle arrest during the early stages of cellular damage, safeguarding cells against DNA damage [51, 52]. These cell-cycle arrest biomarkers have been extensively validated as early kidney injury biomarkers [16, 17]. Although small studies have investigated their use in predicting AKI in COVID-19, the results have been inconsistent [53, 54]. Weiss et al., in a multicenter study, found a high predictive value of urinary [TIMP-2] x [IGFBP7] for moderate or severe AKI [21]. However, our study differs in several points including a smaller sample size, a greater ARDS severity, the timing of sample collection and the choice of the primary endpoint. Husain-Syed et al., in contrast to our findings, described an association between elevated (TIMP-2) x (IGFBP7) and adverse clinical outcomes in patients with COVID-19 severe ARDS [53].

Our study has several strengths, such as high-quality, prospective data collection, including inflammatory cytokines and biomarkers potentially associated with AKI. However, its limitations include a relatively small sample size, the single-center design, and a restricted inclusion period. Most patients were admitted after July 2020 and, therefore, received corticosteroids, intensified anticoagulation, and delayed intubation. Some also received anti-IL-6 treatment based on admission CRP levels. The monocentric design raises concerns about the study’s external validity, and results may not necessarily apply to newer SARSCoV-2 variants emerging after our inclusion period. Then, the clustering analyses was achieved only on the first imputed dataset. Consequently, we did all the remining analysis on that unique dataset. This method might have overlooked the variability introduced by the imputation process and could have led to biased estimates and incorrect inferences. Finally, our binary tree was tested exclusively within our study population and requires validation in external populations.

## Conclusions

In summary, our study documents the presence of distinct kidney outcomes within a population of critically ill patients with severe COVID-19 pneumonia. Several biomarkers show promise in identifying these unique patient clusters. Our findings underscore the heterogeneity of this patient population, which should be considered when interpreting previous study results and designing future trials for critically ill patients with severe COVID-19 pneumonia.

## Supporting information

S1 Appendix Contains all the supporting information files and figures. (DOCX)

S1 Data Contains all data used in the study. (XLSX)

## List of abbreviations

AKD: acute kidney disease
AKI: acute kidney injury
ARDS: acute respiratory distress syndrome
BMI: body mass index
C-AKI: COVID-19 related acute kidney injury
CKD: chronic kidney disease
CRP: C-reactive protein
eGFR: glomerular filtration rare
EO-AKI: early-onset AKI
HCPC: : hierarchical clustering on principal components
ICU: intensive care unit
IGFBP-7: insulin-like growth factor-binding protein 7
IL: interleukin
KDIGO: Kidney Disease: Improving Global Outcomes
L-FABP: liver-type fatty acid-binding protein
MAKE: major adverse kidney events
MDRD: modification of diet in renal disease
mHLA-DR: human histocompatibility leukocyte antigen expression rate (HLA) -DR on blood monocytes
PCT: : procalcitonin
RRT: renal replacement therapy
SCr: serum creatinine
SAPSII: simple acute physiology score 2
sRAGE: soluble receptor for advanced glycation end products
suPAR: soluble urokinase plasminogen activator receptor
TIMP-2: tissue inhibitor of metalloproteinase 2
