# Supplementary material for: Clustering based on renal and inflammatory admission parameters in critically ill patients admitted to the ICU
Source: PLoS One. 2024 Nov 1;19(11):e0307938. doi: 10.1371/journal.pone.0307938 (PMC11530013; doi:10.1371/journal.pone.0307938)
Supplement: S1 Appendix — (DOCX) [file pone.0307938.s001.docx]

**Supporting Information**

**Cluster based on renal and inflammatory parameters on admission in critically ill patients admitted in ICU.**

**Definitions**

AKI diagnosis and staging were defined according to the classification of Kidney Disease: Improving Global Outcomes (KDIGO) (1), using only the serum creatinine (SCr) component. “Baseline SCr” was the best outpatient SCr value between 7 and 365 days before ICU admission or, if unavailable, was estimated using the Modification of Diet in Renal Disease (MDRD) equation (1). Early-onset AKI (EO-AKI) was defined as AKI occurring within 7 days after ICU admission: only the first EO-AKI episode was taken into account in the study. Patients were stratified according to the highest AKI stage attained during this first episode.

EO-AKI recovery was based on a sustained (≥48hours) and complete reversal of AKI by KDIGO criteria and therefore a minimum of 48 hours of renal recovery was necessary to separate two distinct AKI episodes. EO-AKI was classified as transient, persistent and AKD according to the *Acute Dialysis Quality Initiative* (2). “Transient” AKI was defined as renal recovery within 48 h of AKI onset, and “persistent” AKI as renal recovery occurring ≥3 days and <7 days of EO-AKI onset. Acute kidney disease (AKD) was characterized when AKI stage 1 or greater persisted ≥7 days after EO-AKI onset (2) (Annex 2). Chronic kidney disease (CKD) staging was defined according to the KDIGO criteria (1).

1. Kellum JA, Lameire N, KDIGO AKI Guideline Work Group. Diagnosis, evaluation, and management of acute kidney injury: a KDIGO summary (Part 1). Crit Care Lond Engl. 4 févr 2013;17(1):204.

2. Chawla LS, Bellomo R, Bihorac A, Goldstein SL, Siew ED, Bagshaw SM, et al. Acute kidney disease and renal recovery: consensus report of the Acute Disease Quality Initiative (ADQI) 16 Workgroup. Nat Rev Nephrol. avr 2017;13(4):241‑57.

**Laboratory results**

Plasma suPAR levels were measured using commercial Enzyme-linked Immunosorbent Assay (ELISA) kits (suPARnostic® AUTO Flex ELISA Virogates, Copenhagen, Denmark). Plasma sRAGE was measured over human ELISA kits (PROTEIN SIMPLE – BioTechne, Minneapolis, MN, USA). L-FABP concentrations were measured in urine using the ELISA kit from Hycult Biotech (HK404-01, Uden, Netherlands). TIMP-2 and IGFBP7 were measured in urine with the NephroCheckTM Test (Astute Medical, San Diego, CA, USA). The NephroCheck Test is a point-of-care test which was developed to simultaneously measure urine [TIMP-2]*[IGFBP7], whereas [TIMP2]*[IGFBB7] indicates the multiplication of both biomarkers. The quantitative expression of mHLA-DR was determined with the anti-HLA-DR/anti-Monocyte QuantiBRITE assay (BD Biosciences, San Jose, CA, USA). Human pro-inflammatory cytometric bead array (CBA) was used. The human pro-inflammatory cytokine kit simultaneously detects IL-6, IL-10 and CXCL8 cytokines in a single sample.

**Figures and Tables**


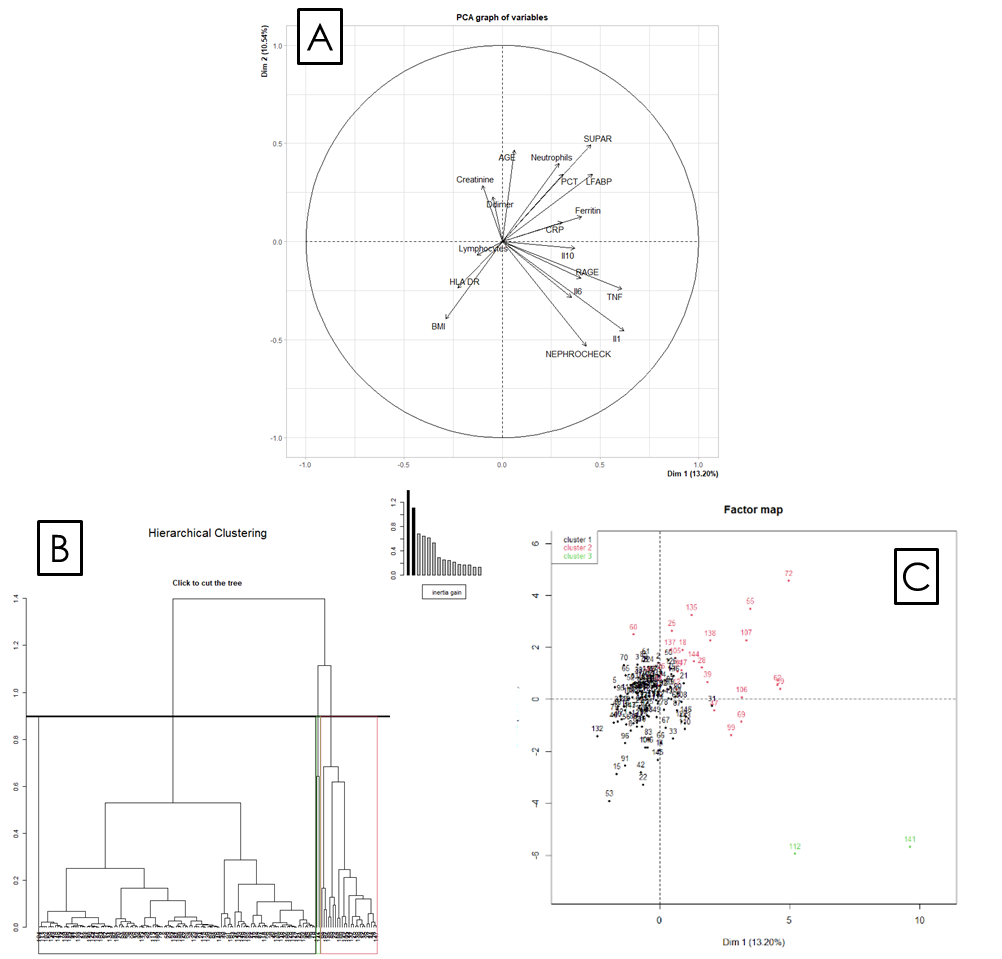


**Figure S1 Hierarchical clustering on principal components using principal component analysis**

A: Graph of variables: projection on the first and second dimensions of the covariates used for the clustering

*B: Dendrogram of ascending hierarchical clustering analysis. Dendrogram obtained after application of hierarchical clustering analysis. The vertical axis of the dendrogram represents the distance between clusters. The horizontal vertical axis represents the patients and clusters. Each junction between two clusters is represented on the graph by the split of a vertical line into two vertical lines. The vertical position of the split, shown by the short horizontal bar gives the distance between the two clusters. The red line shows the cut level that determines the number of clusters.*

*C: Representation of the patients into the first two dimensions. Axes correspond to the first and second dimension of MCA. All patients were represented by their individual coordinates in these dimensions.*

*MCA: multiple correspondence analysis, HC: hierarchical clustering, all patients were represented by their individual coordinates in these dimensions.*

**S1 Table: Description of the included patients and comparison according to the different clusters**

| Parameter | All | Cluster 1 | Cluster 2 | Cluster 3 | pval all | pval 1 vs 2 |
| --- | --- | --- | --- | --- | --- | --- |
| Number of patients | 149 | 122 | 25 | 2 | . | . |
| Age, yr | 71.4 [64.4 ; 76.2] | 70.95 [64.46 ; 75.8] | 74.59 [59.9 ; 79.46] | 66.62 [59.17 ; 74.06] | 0.458 | 0.246 |
| Sex, no (%), men | 108 (72.4) | 88 (72.131) | 19 (76) | 1 (50) | 0.716 | 0.692 |
| BMI, kg/m² | 28.6 [25.4 ; 32] | 28.8 [25.7 ; 32.0] | 27.2 [23.4 ; 32.3] | 29.04 [23.2 ; 34.8] | 0.518 | 0.253 |
| Comorbidities |  |  |  |  |  |  |
| BMI > 30 kg/m² | 67 (45) | 56 (45.902) | 10 (40) | 1 (50) | 0.855 | 0.589 |
| Cardio-vascular disease | 27 (18.2) | 20 (16.393) | 7 (28) |  | 0.312 | 0.172 |
| Chronic respiratory disease | 7 (4.6) | 7 (5.738) |  |  | 0.444 | 0.220 |
| Immunomodulatory treatments | 24 (16.2) | 18 (14.754) | 5 (20) | 1 (50) | 0.342 | 0.511 |
| Diabetes mellitus | 24 (16.2) | 18 (14.754) | 5 (20) | 1 (50) | 0.342 | 0.511 |
| Charlson score | 1 [0 ; 3] |  | 1 [1 ; 3] | 10 [8 ; 12] | 0.047 | 0.560 |
| Time from 1^st^ symp to ICU admission | 9 [7 ; 11] | 9 [7 ; 11] | 11 [7 ; 13] | 3 [3 ; 3] | 0.034 | 0.117 |
| Converting Enzyme Inhibitors | 4 (2.6) | 3 (2.459) | 1 (4) |  | 0.885 | 0.666 |
| Diarrhea | 11 (7.4) | 11 (9.016) |  |  | 0.269 | 0.119 |
| Lopinavir Ritonavir | 2 (1.4) | 1 (0.82) | 1 (4) |  | 0.447 | 0.211 |
| Remdesivir | 47 (31.6) | 43 (35.246) | 3 (12) | 1 (50) | 0.064 | 0.022 |
| Corticosteroids | 130 (87.2) | 111 (90.984) | 17 (68) | 2 (100) | 0.006 | 0.002 |
| Antimicrobial therapy | 36 (24.2) | 25 (20.492) | 10 (40) | 1 (50) | 0.080 | 0.037 |
| Aminoglycosides | 2 (1.4) | 1 (0.82) | 1 (4) |  | 0.447 | 0.211 |
| Vancomycine | 2 (1.4) | 1 (0.82) | 1 (4) |  | 0.447 | 0.211 |
| Diuretics | 132 (88.6) | 109 (89.344) | 21 (84) | 2 (100) | 0.655 | 0.447 |
| Extension of COVID lesion on CT scan | 47 [33 ; 63] | 46.5 [30 ; 60] | 58 [46 ; 70] | 39 [33 ; 45] | 0.044 | 0.018 |
| *Type of variant- Clade\|Lineage\|WHOlabel* |  |  |  |  |  |  |
| 19B\|A | 1 (0.67) | 0 | 1 (4.55) | 0 |  |  |
| 20A\|B.1.160\|EU2 | 59 (39.60) | 47 (41.59) | 10 (45.46) | 2 (100) | 0.83 | 0.51 |
| 20A\|otherslineagesthan | 13 (8.72) | 11 (9.74) | 2 (9.09) |  | . |  |
| 20C\|B.1.367 | 1 (0.67) | 1 (0.89) |  |  | . |  |
| 20D\|B.1.1.16 | 1 (0.67) | 1 (0.89) |  |  | . |  |
| 20E\|B.1.177\|EU1 | 10 (6.7) | 8 (7.08) | 2 (9.09) |  | . |  |
| 20I\|B.1.1.7\|Alpha | 2 (1.34) | 2 (1.77) |  |  | . |  |
| nongenotyped | 43 (38.053) | 7 (31.82) |  | . |  |  |
| *During ICU first two days* |  |  |  |  |  |  |
| SAPS II | 35 [29 ; 44] | 35 [29 ; 42] | 42 [31 ; 49] | 45.5 [45 ; 46] | 0.033 | 0.030 |
| SOFA | 4 [3 ; 5] | 4 [3 ; 5] | 5 [4 ; 8] | 10 [5 ; 15] | 0.004 | 0.003 |
| PaO2/FiO2 | 101 [70 ; 232] | 106 [76 ; 280] | 76 [58 ; 104] | 112 [102 ; 123] | 0.048 | 0.015 |
| Vasopressors | 18 (12) | 10 (8.197) | 7 (28) | 1 (50) | 0.006 | 0.005 |
| Invasive mechanical ventilation | 20 (13.4) | 12 (9.836) | 7 (28) | 1 (50) | 0.016 | 0.014 |
| Renal replacement therapy | 2 (1.4) |  | 2 (8) |  | 0.002 | 0.002 |
| Pneumonia on admission | 11 (7.4) | 9 (7.377) | 1 (4) | 1 (50) | 0.057 | 0.541 |
| Bacteriemia | 4 (2.6) | 1 (0.82) | 3 (12) |  | 0.007 | 0.002 |
| *Characteristics of AKI during ICU stay* |  |  |  |  |  |  |
| No AKI | 103 (69.1) | 92 (75.41) | 11 (44) |  | 0.002 | 0.008 |
| Transient AKI | 20 (43.4) | 14 (11.475) | 6 (24) |  | . | . |
| Persistant AKI | 12 (26) | 6 (4.918) | 5 (20) | 1 (50) | . | . |
| Acute kidney disease | 14 (30.4) | 10 (8.197) | 3 (12) | 1 (50) | . | . |
| No AKI | 103 (69.2) | 93 (76.23) | 10 (40) |  | <0.001 | 0.002 |
| Kdigo 1 | 27 (18.2) | 19 (15.574) | 8 (32) |  | . | . |
| Kdigo 2 | 9 (6) | 4 (3.279) | 4 (16) | 1 (50) | . | . |
| Kdigo 3 | 10 (6.8) | 6 (4.918) | 3 (12) | 1 (50) | . | . |
| *During the whole ICU stay* |  |  |  |  |  |  |
| Vasopressors | 50 (33.6) | 35 (28.689) | 13 (52) | 2 (100) | 0.011 | 0.024 |
| Invasive mechanical ventilation | 50 (33.6) | 36 (29.508) | 12 (48) | 2 (100) | 0.027 | 0.072 |
| ECMO | 1 (0.6) | 1 (0.82) |  |  | 0.895 | 0.650 |
| Invasive mechanical ventilation duration | 0 [0 ; 5] | 0 [0 ; 2] | 0 [0 ; 11] | 8 [6 ; 10] | 0.029 | 0.051 |
| Pulmonary embolism | 8 (5.4) | 7 (5.738) | 1 (4) |  | 0.888 | 0.727 |
| Ventilator associated pneumoniae | 18 (12) | 13 (10.656) | 5 (20) |  | 0.371 | 0.194 |
| Renal replacement therapy | 21 (14) | 15 (12.295) | 5 (20) | 1 (50) | 0.204 | 0.306 |
| Decision not to intubate during ICU Stay | 25 (16.8) | 16 (13.115) | 7 (28) | 2 (100) | 0.001 | 0.062 |
| Length of ICU stay, d (IQR) | 7 [5 ; 12] | 7 [5 ; 11] | 9 [6 ; 15] | 10 [7 ; 13] | 0.197 | 0.090 |
| Death in ICU | 45 (30.2) | 30 (24.59) | 13 (52) | 2 (100) | 0.002 | 0.006 |
| Length of Hospital Stay, d (IQR) | 15 [10 ; 24] | 16 [10 ; 24] | 15 [9 ; 21] | 10 [7 ; 13] | 0.464 | 0.629 |
| In-hospital death | 52 (34.8) | 35 (28.689) | 15 (60) | 2 (100) | 0.002 | 0.003 |
| RRT at hospital discharge (missing=59) | 1 (1.2) | 9 (11.538) | 1 (12.5) |  | 0.936 | 0.936 |
| GFR < 60 mL/min at 90 days (missing=63) | 10 (11.6) | 1 (1.235) |  |  | 0.737 | 0.737 |
| RRT at 90 days (missing= 62) | 0 | 0 | 0 | 0 |  |  |
| Death at 90 days | 55 (37) | 37 (30.328) | 16 (64) | 2 (100) | 0.001 | 0.001 |
| MAKE-90 | 65 (43.6) | 46 (37.705) | 17 (68) | 2 (100) | 0.006 | 0.005 |

*AKI: Acute kidney injury, BMI: Body mass index, ECMO : Extracorporeal membrane oxygenation, GFR : Glomerular filtration rate, ICU : Intensive care unit, KDIGO: Kidney Disease Improving Global Outcome) MAKE-90 : Major advert kidney event at day 90, RRT : Renal replacement therapy, SAPS II : Simple acute physiology score II, SOFA : Sequential organ failure assessment.*

**S2 Table: Biological characteristics of the different clusters**

| Parameter | All | Cluster 1 | Cluster 2 | Cluster 3 | pval all | pval 1 vs 2 |
| --- | --- | --- | --- | --- | --- | --- |
| Number of patients | 149 | 122 | 25 | 2 |  |  |
| Neutrophils^a^ | 7 [5 ; 10.6] | 6.47 [4.76 ; 9.32] | 12.82 [9.46 ; 17.02] | 8.42 [2.4 ; 14.44] | <0.001 | <0.001 |
| Lymphocytes^a^ | 0.6 [0.4 ; 1] | 0.64 [0.46 ; 0.87] | 0.82 [0.47 ; 1.11] | 0.63 [0.4 ; 0.86] | 0.223 | 0.086 |
| Procalcitonin^b^ *(miss=6)* | 0.2 [0.2 ; 0.6] | 0.2 [0.11 ; 0.38] | 0.58 [0.26 ; 1.79] | 0.65 [0.11 ; 1.18] | <0.001 | <0.001 |
| C Reactive Protein^c^ *(miss=32)* | 128 [74 ; 176] | 129 [82 ; 143] | 129 [110 ; 217] | 175.5 [129 ; 222] | 0.046 | 0.023 |
| D-dimers^d^ | 1252 [786 ; 2246] | 1211 [754 ; 2012] | 1916 [953 ; 3381] | 1571 [1216 ; 1926] | 0.265 | 0.119 |
| Fibrinogen^e^ *(miss=1)* | 7.4 [6.2 ; 8] | 7.1 [6.1 ; 7.9] | 8 [7.5 ; 8.8] | 7.55 [6 ; 9.1] | 0.020 | 0.005 |
| Ferritin^b^ *(miss=5)* | 1126 [635 ; 1944.6] | 1029.5 [524 ; 1677] | 1466 [789 ; 2135] | 1327.5 [226 ; 2429] | 0.076 | 0.023 |
| Il10^f^ *(miss=1)* | 3.4 [2 ; 5.8] | 3.2 [1.85 ; 5.1] | 6.3 [4.2 ; 10.9] | 15.42 [1.93 ; 28.9] | 0.002 | <0.001 |
| Il12^f^ *(miss=1)* | 0.2 [0 ; 1.4] | 0.2 [0 ; 1] | 1.13 [0 ; 2] | 22.15 [3.3 ; 41] | 0.002 | 0.011 |
| Il1b^f^ *(miss=1)* | 0.2 [0 ; 1] | 0 [0 ; 1] | 0.5 [0 ; 1.83] | 18.75 [5.5 ; 32] | 0.009 | 0.088 |
| Il6^f^ *(miss=1)* | 34 [10.8 ; 67.4] | 24.75 [9.1 ; 57.8] | 82.6 [44.9 ; 121.3] | 2499.7 [18 ; 4981.4] | <0.001 | <0.001 |
| Il8^f^ *(miss=1)* | 25.4 [16.6 ; 36.8] | 23.05 [15.3 ; 32.99] | 37.02 [26.1 ; 53.4] | 387.77 [3.73 ; 771.8] | 0.001 | <0.001 |
| TNF alpha^f^ *(miss=1)* | 0.2 [0 ; 1.2] | 0 [0 ; 1.1] | 1 [0.2 ; 1.4] | 6.7 [4.6 ; 8.8] | 0.002 | 0.008 |
| mHLA DR^g^ (miss=13) | 9487 [6472.6 ; 13337.6] | 9794.5 [7003 ; 13491] | 7774 [4881 ; 9571] | 7790 [7637 ; 7943] | 0.032 | 0.012 |
| mHLA DR/Il6 (miss=13) | 3.6 [1.2 ; 7.8] | 3.09 [0.78 ; 6.3] | 9.2 [5.79 ; 19.76] | 327.27 [2.27 ; 652.27] | <0.001 | <0.001 |
| [TIMP-2] × [IGFBP7] *(miss=2)* | 0.4 [0.2 ; 1] | 0.43 [0.19 ; 1.02] | 0.59 [0.29 ; 1.23] | 8.87 [7.31 ; 10.42] | 0.039 | 0.377 |
| L-FABP/U Creat | 190 [80.4 ; 470] | 176.65 [70.8 ; 326.6] | 402.57 [184.25 ; 1203.39] | 667.9 [153.04 ; 1182.76] | 0.003 | 0.001 |
| sRAGE^h^ *(miss=2)* | 2906 [1460 ; 7156] | 2887.5 [1449 ; 6286] | 2906 [1823 ; 10352] | 16274.5 [3326 ; 29223] | 0.323 | 0.467 |
| suPAR^d^ *(miss=2)* | 6.8 [5.4 ; 9] | 6.27 [5.19 ; 8.09] | 10.05 [8.15 ; 14.41] | 8.28 [7.72 ; 8.83] | <0.001 | <0.001 |

*a: G/L, b: µg/L, c: mg/L, d: ng/mL, e: g/L, f:pg/L, g: mHLA DR, Monocytic human leukocyte antigen-DR*, *IGFBP-7: insulin-like growth factor-binding protein* 7, *Il: Interleukin, LFABP : Liver fatting acid binding protein, sRAGE: Soluble receptor of advences glycation end products, suPAR: Soluble Urokinase Plasminogen Activator Receptor, TIMP-2:* *Tissue inhibitor of metalloproteinase 2,* *TNF: Tumor necrosis factor, U Creat: urinary creatinine*

**Table S3: Factors associated with Cluster 1 or 2**

| Risk factor | OR | IC95 | pvalue | OR | IC95 | pvalue |
| --- | --- | --- | --- | --- | --- | --- |
|  | **Cluster 1** | | | **Cluster 2** | | |
| Symptoms to ICU>10 days | 0.49 | [0.21 ; 1.14] | 0.10 | 2.45 | [1.02 ; 5.87] | 0.04 |
| Age > 70 years | 0.78 | [0.34 ; 1.83] | 0.57 | 1.32 | [0.55 ; 3.16] | 0.54 |
| Sex (Male) | 0.91 | [0.35 ; 2.34] | 0.84 | 1.25 | [0.46 ; 3.38] | 0.67 |
| BMI > 30 kg/m² | 1.23 | [0.53 ; 2.88] | 0.63 | 0.78 | [0.33 ; 1.88] | 0.58 |
| Comorbidities | 0.53 | [0.23 ; 1.26] | 0.15 | 1.60 | [0.67 ; 3.83] | 0.29 |
| Immunodepression | 0.61 | [0.21 ; 1.71] | 0.34 | 1.38 | [0.46 ; 4.13] | 0.56 |
| Lopinavir ritonavir | 0.21 | [0.01 ; 3.55] | 0.28 | 5.13 | [0.31 ; 84.8] | 0.25 |
| Remdesivir | 3.13 | [1.02 ; 9.64] | 0.05 | 0.25 | [0.07 ; 0.88] | 0.03 |
| Corticosteroids | 4.25 | [1.51 ; 11.93] | <0.01 | 0.21 | [0.07 ; 0.59] | <0.01 |
| Antimicrobial therapy | 0.37 | [0.15 ; 0.91] | 0.03 | 2.51 | [1.01 ; 6.24] | 0.05 |
| Aminosides | 0.21 | [0.01 ; 3.55] | 0.28 | 5.13 | [0.31 ; 84.8] | 0.25 |
| Vancomycin | 0.21 | [0.01 ; 3.55] | 0.28 | 5.13 | [0.31 ; 84.8] | 0.25 |
| Diuretics | 1.46 | [0.44 ; 4.88] | 0.54 | 0.61 | [0.18 ; 2.07] | 0.43 |
| SOFA > 4 | 0.38 | [0.16 ; 0.92] | 0.03 | 2.23 | [0.92 ; 5.43] | 0.08 |
| Vasopressors | 0.21 | [0.07 ; 0.61] | <0.01 | 3.99 | [1.37 ; 11.65] | 0.01 |
| IMV | 0.26 | [0.09 ; 0.72] | <0.01 | 3.32 | [1.17 ; 9.44] | 0.02 |
| Pneumonia on admission | 1.00 | [0.2 ; 4.89] | 1.00 | 0.48 | [0.06 ; 3.89] | 0.49 |
| Neutrophils > 8 G/L | 0.13 | [0.04 ; 0.41] | <0.01 | 9.51 | [2.7 ; 33.42] | <0.01 |
| Lymphocytes > 0.6 G/L | 1.64 | [0.68 ; 3.94] | 0.27 | 0.57 | [0.23 ; 1.42] | 0.23 |
| CRP > 100 mg/L | 0.36 | [0.12 ; 1.1] | 0.07 | 2.50 | [0.8 ; 7.77] | 0.11 |
| D-Dimers > 1500 µ/L | 0.60 | [0.26 ; 1.39] | 0.24 | 1.66 | [0.7 ; 3.93] | 0.25 |
| Fibrinogen > 8 g/L | 0.23 | [0.08 ; 0.64] | <0.01 | 5.25 | [1.7 ; 16.18] | <0.01 |
| Ferritin > 1000 µg/L | 0.55 | [0.23 ; 1.32] | 0.18 | 1.93 | [0.78 ; 4.8] | 0.16 |
| PCT > 0.5 µg/L | 0.22 | [0.09 ; 0.52] | <0.01 | 4.51 | [1.83 ; 11.13] | <0.01 |
| Il10 > 4.5 pg/mL | 0.26 | [0.1 ; 0.66] | <0.01 | 4.24 | [1.58 ; 11.35] | <0.01 |
| Il12 > 0.2 pg/mL | 0.29 | [0.11 ; 0.73] | <0.01 | 3.02 | [1.18 ; 7.75] | 0.02 |
| Il1 > 0.2 pg/mL | 0.44 | [0.18 ; 1.03] | 0.06 | 1.94 | [0.81 ; 4.67] | 0.14 |
| Il6 > 34 pg/mL | 0.12 | [0.04 ; 0.37] | <0.01 | 10.50 | [2.98 ; 36.94] | <0.01 |
| Il8 > 25 pg/mL | 0.24 | [0.09 ; 0.64] | <0.01 | 4.70 | [1.66 ; 13.32] | <0.01 |
| TNF > 0.2 pg/mL | 0.27 | [0.11 ; 0.68] | <0.01 | 3.23 | [1.26 ; 8.28] | 0.01 |
| mHLA DR > 9500 pg/mL | 2.37 | [1 ; 5.59] | 0.05 | 0.50 | [0.21 ; 1.19] | 0.12 |
| Il6/mHLA DR (x 1000) > 3.6 | 0.12 | [0.04 ; 0.37] | <0.01 | 10.50 | [2.98 ; 36.94] | <0.01 |
| (TIMP-2) × (IGFBP7) > 0.3 | 0.86 | [0.35 ; 2.14] | 0.75 | 1.01 | [0.4 ; 2.54] | 0.98 |
| LFABP/ creat > 190 | 0.33 | [0.14 ; 0.82] | 0.02 | 3.23 | [1.26 ; 8.28] | 0.01 |
| sRAGE > 2900 pg/mL | 0.80 | [0.35 ; 1.85] | 0.60 | 1.05 | [0.44 ; 2.48] | 0.91 |
| suPAR > 6.8 | 0.05 | [0.01 ; 0.23] | <0.01 | 17.02 | [3.84 ; 75.43] | <0.01 |
| Parenchymal lesions > 75 % | 0.27 | [0.06 ; 1.29] | 0.10 | 4.09 | [0.86 ; 19.55] | 0.08 |

*BMI: Body mass index, CRP: C reative protein, ICU: Intensive care unit, IGFBP-7: insulin-like growth factor-binding protein 7, Il : Interleukin, IM : Invasive mechanical ventilation, LFABP: Liver fatting acid binding protein, mHLA DR, Monocytic human leukocyte antigen-DR, SOFA: Sequential organ failure assessment, sRAGE: Soluble receptor of advances glycation end product, suPAR: Soluble Urokinase Plasminogen Activator Receptor, TIMP-2 : Tissue inhibitor of metalloproteinase 2, TNF : Tumor necrosis factor. Immunosuppression: Hematologic malignancy (active or in remission for less than 5 years), hematopoietic stem cell transplant for less than 5 years, active solid cancer, leukopenia < 1 G/L or neutropenia ≤ 0.5 G/L, solid organ transplantation, syndrome acquired immunodeficiency, long-term corticosteroid therapy ≥ 0.5 mg/kg/day of prednisone equivalent for at least 3 weeks, immunosuppressive or immunomodulatory treatment*

Figure S2: Prediction to belong to Cluster 1 or Cluster 2 using Random Forest algorithm

*%Inc MS:, per cent increase in mean squared error, CRP: C reative protein, Il: interleukin,; LFABP: liver fatty acid binding protein, MCA: multiple correspondence analysis, mHLA DR, Monocytic human leukocyte antigen-DR, PCT: procalcitonin, SUPAR: soluble urokinase plasminogen activator receptor, TNF: tumor necrosis factor*

**Figure S3: prediction to belong to cluster 2 – CART**


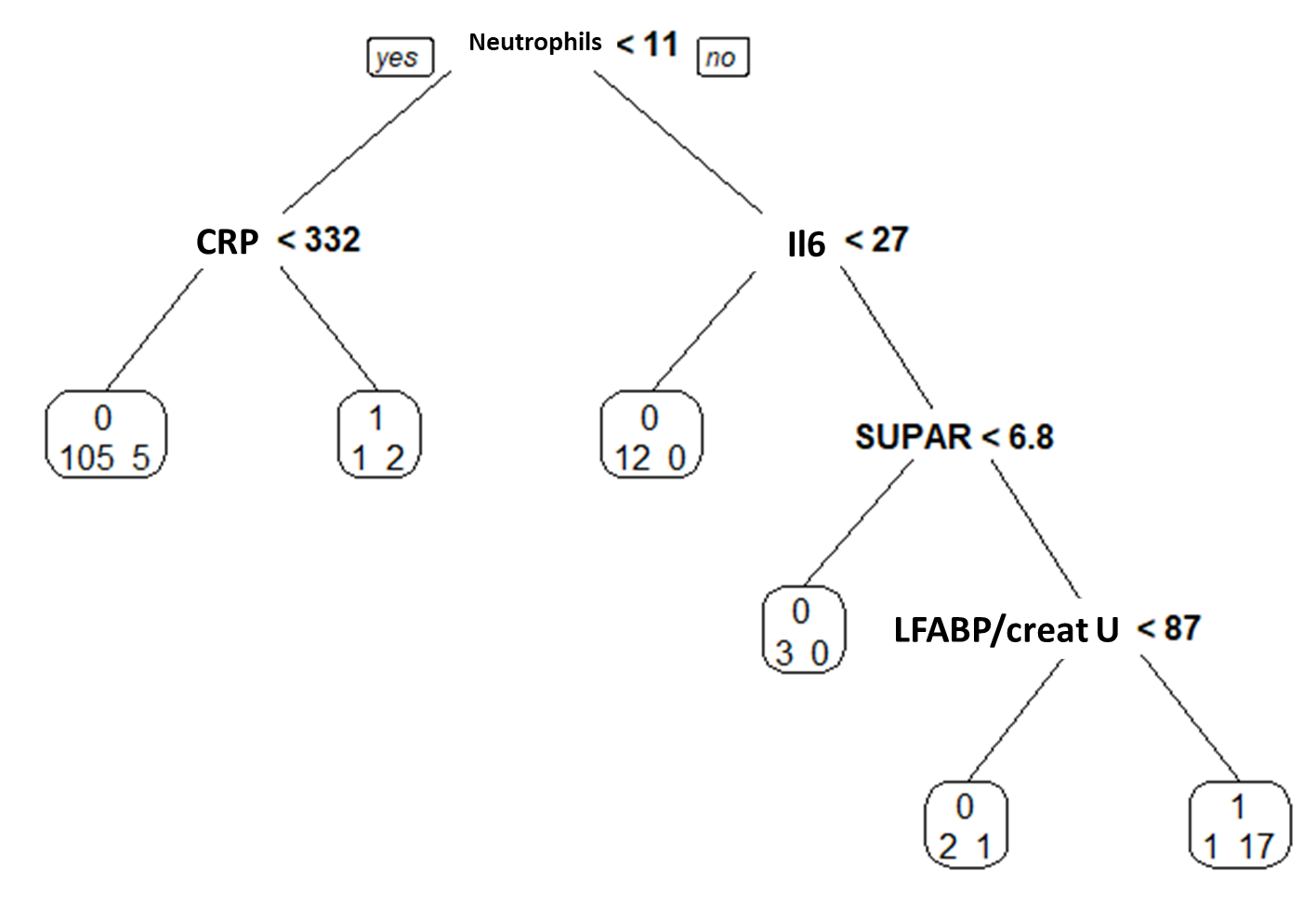


*Tree is built such as 0: should not belong to cluster 2, 1: should belong to cluster 2; and below: left number actual patients in the studied population who did not belong to cluster 2; right number: actual patients in the studied population who belonged to Cluster 2.*

*CRP: C-reactive protein (in mg/L), creat U: urinary creatinine (in mmol/L), Il, interleukin (in pg/L,; LFABP, liver fatty acid binding protein (in ng/mL,; SUPAR, soluble urokinase plasminogen activator receptor (in ng/mL)*

*The binary tree was built in the training set using Breiman methods with Rpart package version 4.1-10, R version 3.1.0. The structure is similar to a real tree, from the bottom up: there is a root, where the first split happens. After each split, two new nodes are created. Each node contains only a subset of the patients. The partitions of the data, which are no longer split, are called terminal nodes or leafs. The second stage of the procedure consists in pruning the tree using cross-validation. Pruning means to shorten the tree, which makes trees more compact and avoids over-fitting to the training data. Each split is examined if it makes a reliable improvement. The six variables used by the binary tree are neutrophils, CRP, IL-6, SUPAR and LFABP/urinary creatinine ratio. The accuracy of the binary tree evaluated in the training dataset is given in Table 5*

**Table S4: Distribution of patients based on decision tree**

| **Cluster** | **Should not belong to Cluster 2** | **Should belong to Cluster 2** |
| --- | --- | --- |
| No Cluster 2 | 122 (98%) | 2 (2%) |
| Cluster 2 | 6 (24%) | 19 (76%) |

**Table S5: Association with AKI on admission and with ICU death, univariate logistic regression**

| Cluster | Odd ratio | IC95 | pvalue |
| --- | --- | --- | --- |
| Association with AKI on admission | | | |
| Cluster1 | 0.235 | [0.09 ; 0.59] | <0.01 |
| Cluster2 | 4.945 | [1.93 ; 12.67] | <0.01 |
| Association with ICU mortality | | | |
| Cluster1 | 0.261 | [0.11 ; 0.62] | <0.01 |
| Cluster2 | 3.115 | [1.29 ; 7.52] | 0.01 |
| Association with 90-day mortality | | | |
| Cluster1 | 0.218 | [0.09 ; 0.53] | <.01 |
| Cluster2 | 3.873 | [1.57 ; 9.53] | <.01 |
| Association with MAKE-90 | | | |
| Cluster1 | 0.255 | [0.1 ; 0.63] | <.01 |
| Cluster2 | 3.364 | [1.35 ; 8.4] | <.01 |

*AKI: Acute kidney injury, ICU: Intensive Care unit, MAKE: Major acute kidney event.*

**Figure S4: Predictors of death and MAKE 90 - Random Forest**

*Contribution of each variable to the risk of death (A) and MAKE 90 (B)*

*%Inc MSE: per cent increase in mean squared error, CV: cardiovascular, Il: interleukin, KDIGO: acute kidney injury classification, LFABP: liver fatty acid binding protein, RAGE: receptor of advanced glycation end product, SUPAR: soluble urokinase plasminogen activator receptor, TNF: tumor necrosis factor, SAPSII: simple acute physiology score II,*
